# The predictive value of models of neuromuscular disorders to potentiate clinical translation

**DOI:** 10.1242/dmm.049788

**Published:** 2022-08-01

**Authors:** Maaike van Putten

**Affiliations:** Leiden University Medical Center, Department of Human Genetics, 2333 ZC Leiden, The Netherlands

## Abstract

Neuromuscular disorders (NMDs) are a heterogenous group of rare inherited diseases that compromise the function of peripheral nerves and/or muscles. With limited treatment options available, there is a growing need to design effective preclinical studies that can lead to greater success in clinical trials for novel therapeutics. Here, I discuss recent advances in modelling NMDs to improve preclinical studies as well as two articles from this issue that work in parallel to enable a deeper understanding of a particularly rare NMD, known as X-linked myotubular myopathy.

## Abstract (precis)

**Summary:** This Editorial discusses advances in modelling neuromuscular disorders to improve the translatability of preclinical studies. It also highlights two articles from this issue of DMM that provide a deeper understanding of X-linked myotubular myopathy.

## Introduction

Neuromuscular disorders (NMDs) encompass a broad collection of rare conditions that impair the functionality of muscles – either directly by affecting muscle fiber integrity or indirectly through defects of the peripheral nervous system or neuromuscular junctions. Historically, classification of NMDs is based on the age of onset and clinical manifestation, such as limb–girdle muscular dystrophy and congenital muscular dystrophy. Inclusion of the mode of inheritance enabled further subdivision of clinically heterogenous disorders (Mercuri et al., 2019). The identification of hundreds of causative genes, catalogued in the Neuromuscular Disorders Gene Table (http://www.musclegenetable.fr/), highlights the genetic diversity of NMDs. In the last decade, state-of-the-art molecular investigations of cases lacking a genetic diagnosis have led to the discovery of even more causative genes, thereby adding to the genetic complexity of, for instance, limb–girdle muscular dystrophy (Straub et al., 2018) and facioscapulohumeral muscular dystrophy (Lemmers et al., 2012).

The emergence of disease specific standards of care has improved the quality of life and life expectancy for several NMDs. However, despite decades of research, commercially available treatment options are, unfortunately, still limited. This most likely results from the genetic complexity, heterogeneity in disease severity and rarity of these disorders. In the last decade, multiple therapeutic strategies have advanced to clinical trials being successful for several NMDs, including spinal muscular atrophy for which three drugs are currently approved (Dowling et al., 2017). Unfortunately, the majority of clinical trials fail owing to insufficient drug exposure to the target nerves and/or muscles, as well as low treatment efficacy. Successful clinical translation largely depends on the quality and translatability of preclinical studies and the predictive value of experimental models, from advanced *in vitro* systems to large animals. Reliable preclinical models are of utmost importance to studying NMDs because of the rarity of these disorders (van Putten et al., 2020). Untangling the genetic complexity of NMDs, and improving the quality and translatability of preclinical studies for novel treatments are main goals of the NMD field, which are being prioritized to ultimately benefit patient diagnosis and treatment.

**Box 1. The natural history of neuromuscular disease models points to the future of disease modelling**

In this issue of DMM, two articles aim to increase our understanding of the natural disease history of the myotubularin (*Mtm1*) KO mouse for XLMTM. The severe and progressive disorder XLMTM is caused by mutations in the *myotubularin 1* (*MTM1*) gene that results in loss of expression and/or function of the MTM1 protein. The *Mtm1* KO mouse is widely used to study XLMTM but a lack of knowledge on the disease pathomechanisms and natural disease history has hampered preclinical investigations. The articles by Sarikaya et al. (2022) and Buono et al. (2022) provide comprehensive longitudinal natural history data of the *Mtm1* KO mouse. Through assessments of muscle functionality and histopathology, and proteomics and transcriptomics of a wide variety of skeletal muscles, they provide the field with vital information on disease pathology throughout the lifespan of the model. Furthermore, Buono and colleagues confirmed the applicability of the *Mtm1* KO mouse for studying the therapeutic effects of *Dnm2* reduction by using antisense oligonucleotides. These articles also identify which functional, histological and molecular outcome measures are most appropriate for future preclinical trials. This is likely to improve the quality of future preclinical studies for XLMTM and increase the predictability of success of therapeutic approaches in clinical settings.

## Advances **of** *in vitro* drug testing

Availability of cell models that recapitulate the molecular complexity of NMDs are essential for proof-of-principle studies and compound selection before advancing to animal models. Traditionally, immortalized cells of human or murine origin were used most often. More recently, induced pluripotent stem cell technology allowed reprogramming of patient- and control-derived cells into 2D and 3D muscle cultures. These developments facilitated direct testing of drug candidates in functional muscle bundles (Wang et al., 2019) and cardiomyocyte cultures (Gartz et al., 2020), enabling simultaneous assessments of treatment efficacy, such as gene correction and protein restoration, and resulting in potential improvements of contractile properties in a human genetic context. Co-culturing organoids in the form of muscles-on-a-chip with, for instance, neuromuscular junctions and/or blood vessels (Osaki et al., 2020), has greatly enhanced translatability of *in vitro* models (reviewed by Santoso and McCain, 2020). Furthermore, increasing the scale to 96-well plates has allowed high-throughput testing of drug candidates. These advances will improve pre-selection of compounds for progression towards preclinical testing in animal models.

## The importance of high-quality preclinical research

The availability of animal models that partly mimic human disease has greatly increased our understanding of the pathophysiology of NMDs. Decades ago, research depended on the use of naturally occurring animal models or the laborious generation of animal models using homologous recombination. More recently, however, gene editing has facilitated and accelerated the development of models carrying specific mutations, thereby greatly expanding the availability of clinically relevant models. CRISPR/Cas9-mediated gene editing, which enables precise introduction of mutations, has further advanced the generation of animal models, allowing for faster and cheaper production (Hinman et al., 2021; Nitahara-Kasahara et al., 2021). Also, the use of humanized mouse models, carrying the human gene with specific patient-derived causative mutations, has benefited from these developments. These humanized models are better equipped for testing of genetic therapies, using human-specific sequences rather than mouse equivalent sequences, thereby improving the translatability of findings (Aartsma-Rus and Van Putten, 2020).

**Fig. 1.**
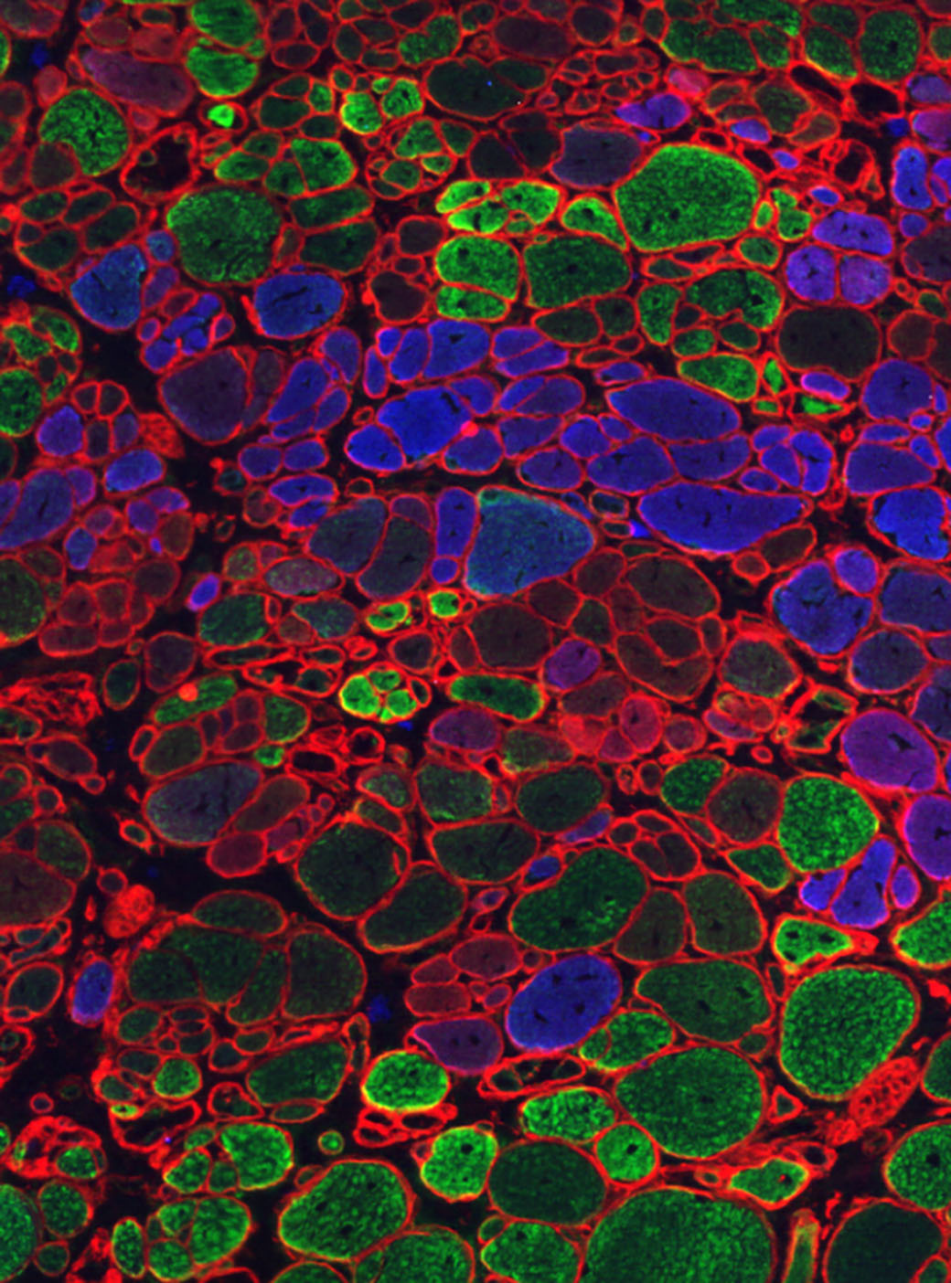
**Fiber-type staining of the tibialis anterior of the mdx-*Utrn*+/- Duchenne muscular dystrophy mouse model at age eight weeks**. Myosin heavy chains type I, IIa and IIb are respectively stained in blue, red and green.

Even though animal models can carry the disease-causing mutation, they do not mimic all aspects of the human disease, and variability in the severity of the disease phenotype exists between them. Exemplified here are animal models for Duchenne muscular dystrophy (DMD). *Mdx* mice have a mutation in the orthologous *Dmd* gene to mimic human DMD and, like DMD patients, they are prone to muscle damage and develop cardiomyopathy later in life. However, these mice are less severely affected owing to very active regeneration, their muscles are not replaced by fat tissue and their life-expectancy is only slightly impeded. Although strategies have been developed to increase disease severity in mouse models, such as the deletion of additional genes like *utrophin* (*Utrn*) (Deconinck et al., 1998) (Fig. 1) or *cytidine monophosphate-N-acetylneuraminic acid hydroxylase* (*Cmah*) (Chandrasekharan et al., 2010), larger animal models, i.e. the DMD rat (Szabó et al., 2021), dog (Hakim et al., 2021) and pig (Stirm et al., 2021), seem to better recapitulate these aspects of human disease (McGreevy et al., 2015). Smaller animal models, such as zebrafish, *Drosophila* and *C. elegans* are advantageous for high-throughput drug screening (Wasala et al., 2020), and are important models for mechanistic and functional studies. However, they do not fully recapitulate the human pathophysiology. For instance, *Drosophila* has a single orthologue for human dystrophin and utrophin genes (Greener and Roberts, 2000) and *C. elegans* lacks an immune system (Ermolaeva and Schumacher, 2014). This heterogeneity across species highlights the need to select the most-suitable model(s) and adds to the complexity of translating preclinical findings to the clinic.

Even though animal models do not fully recapitulate the human pathology, they are essential for assessing drug safety and efficacy, and to help prioritize compounds for further clinical development. With the success rate of clinical trials largely depending on well-designed and reliable preclinical studies that use predictable models, the field has invested particular attention in increasing the quality of these studies. First, awareness was raised for the importance of natural disease history data for disease models. Natural history data provide insights regarding the age of disease onset and disease progression throughout life in the absence of therapeutic interventions. These data are vital to allow better design of preclinical studies, only assessing meaningful outcome measures, of which a large therapeutic window is expected between wildtype and diseased models. The ‘Of Mice and Measures’ project initiated by the DMD field, is an exemplary international effort from researchers, industry and patient organisations, which aims to collect all available natural history data of a novel DMD model, the D2/*mdx* mouse (Gordish-Dressman et al., 2018). This initiative launched a large systematic natural history study that is currently ongoing in two independent laboratories, aimed to directly compare muscle function and pathology of the classic BL10-*mdx* model (on a C57BL/10ScSnJ genetic background) with the D2/*mdx* model (on a DBA/2J genetic background). General consensus on the importance of the availability of natural history data for other NMDs is highlighted by the increasing number of publications on this topic, including two articles on X-linked myotubular myopathy (XLMTM) mouse models (Box 1) in this issue of DMM (Buono et al., 2022; Sarikaya et al., 2022). Second, to enhance scientific rigor and comparability of results between preclinical studies, the global organisation TREAT-NMD (https://treat-nmd.org/) has initiated and facilitated the generation of several standard operating procedures (SOPs) for cell and animal studies for NMDs, such as DMD, spinal muscular atrophy and congenital muscular dystrophy (Grounds et al., 2008; Van Putten et al., 2018; Willmann et al., 2015). Last, compliance with the ARRIVE guidelines, aimed to improve quality of the documentation of preclinical study details, is instrumental (https://arriveguidelines.org/). Taken together, the availability of well-characterized animal models, as well as compliance with efforts to reduce experimental variation and to increase comparability between studies will benefit evaluation of novel therapeutic strategies.

## The road to therapy and future outlook

In recent years, the NMD field has greatly advanced by acknowledging the importance of detailed genetic screening, the collection of natural history data in patient populations, and the availability of informative outcome measures and biomarkers able to assess treatment efficacy in clinical trials (Straub and Bertoli, 2016; Voermans et al., 2021). To facilitate the critical evaluation of preclinical drug studies aimed to de-risk clinical development of therapies for NMDs, the TREAT-NMD Advisory Committee for Therapeutics (TACT) was initiated in 2009 (www.treat-nmd.com/tact). This international multi-disciplinary group of expert researchers and representatives of patient organisations provides guidance to applicants in their therapeutic development path, and has reviewed over 60 programs to date (Heslop et al., 2015; Wagner et al., 2020; Willmann et al., 2020). This has greatly improved the chances of success for new drugs for NMDs in clinical trials and avoided initiation of trials for compounds that have limited preclinical efficiency. Unfortunately, despite these efforts, unforeseen events, such as recent patient deaths associated with AAV-based gene therapies for both X-linked myotubular myopathy and DMD, have occurred in clinical trials (Wilton-Clark and Yokota, 2022). This underlines that, whereas model systems may guide clinical trial design, they are never fully predictive of the human disease, and new therapeutic approaches bring risks when they are first tested in humans. As such, each preclinical and clinical trial – regardless of its results – will improve our knowledge of NMDs and move the field a step closer to potential therapies.

Owing to the limited treatment options for NMDs, there is an urgency to thoroughly understand this heterogenous group of diseases. By exploring disease mechanisms in a range of model systems, from advanced *in vitro* platforms to varied animal models, we can bridge the gap between basic science and the clinic. Disease Models & Mechanisms encourages cutting-edge research in the NMD field. In this issue, we highlight two articles that explore murine XLMTM models with the aim of enabling more-advanced research capabilities for this rare NMD (Box 1). These studies will be joining a growing subject collection of articles dedicated to NMD (https://journals.biologists.com/dmm/collection/37/Neuromuscular-Disease-Models) that follows on from our 2020 Special Issue, which was committed to supporting this evolving and important field of human disease research.
